# Classifiers for Ischemic Stroke Lesion Segmentation: A Comparison Study

**DOI:** 10.1371/journal.pone.0145118

**Published:** 2015-12-16

**Authors:** Oskar Maier, Christoph Schröder, Nils Daniel Forkert, Thomas Martinetz, Heinz Handels

**Affiliations:** 1 Institute of Medical Informatics, University of Lübeck, Lübeck, Germany; 2 Graduate School for Computing in Medicine and Live Science, University of Lübeck, Lübeck, Germany; 3 Institute for Neuro- and Bioinformatics, University of Lübeck, Lübeck, Germany; 4 Department of Radiology and Hotchkiss Brain Institute, University of Calgary, Calgary, Canada; College of Mechatronics and Automation, National University of Defense Technology, CHINA

## Abstract

**Motivation:**

Ischemic stroke, triggered by an obstruction in the cerebral blood supply, leads to infarction of the affected brain tissue. An accurate and reproducible automatic segmentation is of high interest, since the lesion volume is an important end-point for clinical trials. However, various factors, such as the high variance in lesion shape, location and appearance, render it a difficult task.

**Methods:**

In this article, nine classification methods (e.g. Generalized Linear Models, Random Decision Forests and Convolutional Neural Networks) are evaluated and compared with each other using 37 multiparametric MRI datasets of ischemic stroke patients in the sub-acute phase in terms of their accuracy and reliability for ischemic stroke lesion segmentation. Within this context, a multi-spectral classification approach is compared against mono-spectral classification performance using only FLAIR MRI datasets and two sets of expert segmentations are used for inter-observer agreement evaluation.

**Results and Conclusion:**

The results of this study reveal that high-level machine learning methods lead to significantly better segmentation results compared to the rather simple classification methods, pointing towards a difficult non-linear problem. The overall best segmentation results were achieved by a Random Decision Forest and a Convolutional Neural Networks classification approach, even outperforming all previously published results. However, none of the methods tested in this work are capable of achieving results in the range of the human observer agreement and the automatic ischemic stroke lesion segmentation remains a complicated problem that needs to be explored in more detail to improve the segmentation results.

## Introduction

The ischemic stroke, one of the leading causes of death and disability worldwide, is triggered by an obstruction in the cerebrovascular system preventing the blood to reach the brain regions supplied by the blocked blood vessel directly. Irreversible damage of the affected brain cells occurs within minutes to hours depending on the existence and characteristics of collateral connections, which may still supply some affected brain regions with reduced blood flow (hypoperfusion). In contrast to these rather acute changes, tissue alterations induced by secondary molecular effects continue for weeks to month. During this time, the patient’s impairment as well as the appearance of the stroke lesion in magnetic resonance imaging (MRI) datasets, which is an established imaging modality for follow-up stroke assessment, fluctuates.

The reliable and reproducible lesion segmentation in follow-up image sequences is of high interest, since the lesion volume is one important imaging end-point for clinical trials. However, the automatic localization and segmentation of ischemic stroke lesions in MRI volumes is not a trivial task, since the lesion shape and location depends on several factors such as time-from-symptom onset, occlusion site, patient-specific differences regarding the vessel anatomy, collateral connections and potential tissue preconditioning due to a coexisting incomplete stenosis [1]. The presence of other white matter hyperintensities (Leukoaraiosis) may furthermore complicate a precise automatic segmentation. Rekik et al. [2] identified a number of common biological- and imaging-dependent challenges that have to be dealt with when segmenting stroke lesion in MRI volumes, including fogging in diffusion weighted (DWI) sequences, the T2 shine through effect and tissue deformations.

Furthermore, Rekik et al. [2] performed a review of non-chronic ischemic stroke lesion segmentation methods. The majority of the 25 reviewed articles describe voxel-based (n = 13) approaches in contrast to image-based (n = 9), atlas-guided (n = 1) and deformable model (n = 2) methods. Only a few of these are fully automatic approaches and none is based on supervised training of a classifier, which may be beneficial for lesion segmentation in mono-modal and especially when employing multi-spectral image sequences.

Chronic stroke lesion segmentation, on the other hand, has been approached with machine learning techniques. For example, Seghier et al. [3] proposed an outlier search with subsequent fuzzy clustering of voxels in T1-weighted (T1w) MRI datasets for segmentation of chronic lesions. Forbes et al. [4] presented a Bayesian multi-spectral hidden Markov model with individual weights for the different MRI sequences. However, their method was only evaluated on a single case. An interesting semi-automatic as well as automatic method can be found in Wilke et al. [5], which takes the special stroke characteristics into account and employs four-class fuzzy-clustering to segment chronic ischemic stroke lesions in T1w MRI volumes. However, it was found that user-interaction is still required to achieve acceptable segmentation results. Mitra et al. [6] approached the problem of chronic lesion segmentation with a combination of Bayesian-Markov random fields and random decision forests (RDF) for voxel-wise classification in multi-spectral MRI volumes with comparatively good results. A most recent work by Chyzhyk et al. [7] proposes active learning for interactive, single-patient segmentation from multi-spectral volumes. In related previous works, we have shown Extra Tree (ET) forests [8] outperform all previously published methods and also obtained acceptable results with support vector machines (SVM) [9], but found the latter time-consuming and difficult to optimize.

As a drawback, most previously presented methods were only evaluated using a limited number of private datasets that are often insufficiently described, which makes a comparison of these methods difficult, if not impossible. This deficiency can partially be attributed to the lack of publicly available non-acute datasets of ischemic stroke lesions with manual ground truth segmentations.

In this work, we evaluate and compare nine popular classification approaches in a fair and direct comparison using a clinically relevant set of MRI images of sub-acute ischemic stroke patients. These approaches include comparably simple methods like k-Nearest-Neighbors (kNN) and Gaussian Naive Bayes (GNB), statistical approaches like Generalized Linear Models (GLM), as well as high-level machine learning techniques like Random Decision Forests (RDF) and Convolutional Neural Networks (CNN). The results shed light on the nature of the segmentation problem and constitute a solid base for developing more specialized solutions. The evaluation includes a juxtaposition of mono- against multi-spectral MRI datasets and takes inter-observer variability into account.

In contrast to our previous work [8], we now investigate a wide range of classifiers, employ a clinically more relevant best-effort appraoch and investigate the influence of multiple raters on the machine learning methods.

## Materials and Methods

### Data and ground truth

Various MRI sequences are typically utilized in the clinical routine for the assessment of ischemic stroke lesions, as they provide insights into different aspects of the disease. Fluid attenuation inversion recovery (FLAIR) MRI is probably the most prominent technique for imaging in sub-acute ischemic stroke patients, followed by DWI and T1w datasets. In the sub-acute phase (here: >24 hours and <2 weeks), the lesion usually appears hyper-intense in FLAIR and DWI and hypo-intense in T1w datasets.

The database used for evaluation in this study consists of 37 cases acquired routinely for two clinical studies on spatial neglect [10–12]. More information on the patients, lesion characteristics, imaging parameters, and image quality are detailed in a previous work [8].

Each dataset was manually segmented (as filled volume) in axial FLAIR images by two observers with several years of dedicated experience in stroke imaging (GTG and GTL). If required and available, other MRI sequences were used to resolve ambiguities. In case of a previous acute ischemic stroke history, only the newest ischemic stroke lesions were segmented. Hemorrhages were only included in the manual lesion segmentations if completely encircled by ischemic tissue.

The pre-processed cases as well as the ground-truth and segmentation results are available from http://dx.doi.org/10.6084/m9.figshare.1585018. Some of the cases have recently been incorporated in the evaluation dataset of the ISLES 2015 Ischemic Stroke Lesion Segmentation challenge (www.isles-challenge.org), together with an larger set of images.

### Image segmentation as voxel classification task

Treating a segmentation problem as voxel-wise segmentation task enables the application of machine learning techniques. Each image voxel is treated as one stand-alone sample, characterized by a number of features (e.g. its gray-value) and assigned to a binary class (0 = background, 1 = lesion). To obtain a generalized solution model for the problem, a classifier is trained on a set of labeled training samples. During the subsequent application, a formerly unseen volume is passed to the trained classifier, which decides for every voxel whether it belongs to an ischemic stroke lesion or not.

### The image features

Four different types of simple image features are employed in this work, namely the *intensity feature*, the *weighted local mean*, the *2D center distance* and the *local histogram*. They provide the classifier with information of the voxel’s gray-value and the gray-values in a small neighborhood as well as their distribution. More details about these features can be found in Maier et al. [8].

### Pre- and post-processing

**Fig 1:**
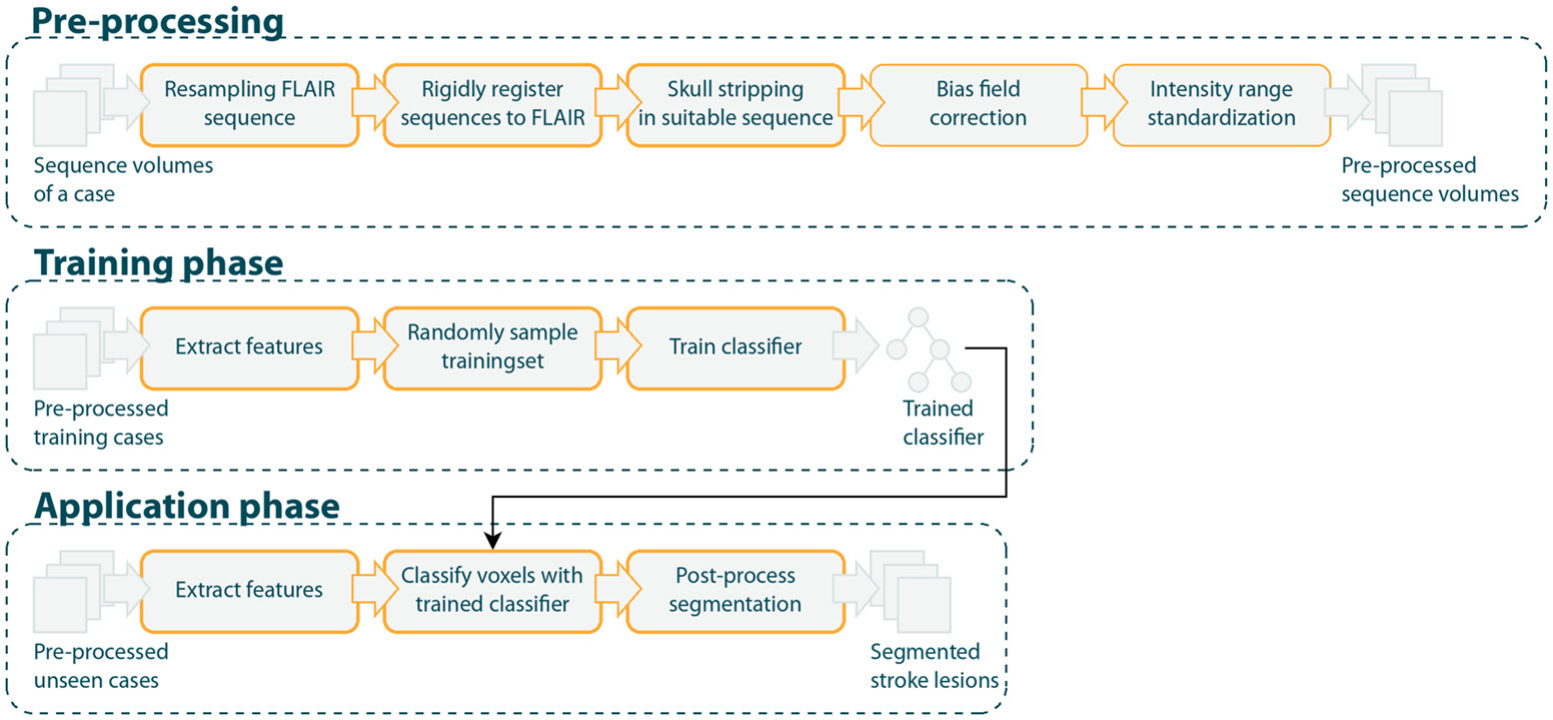
Processing pipeline.

All images, both for the training and testing phase, are prepared using the fully automatic pre-processing pipeline described in Maier et al. [8]. This includes down-sampling, intracranial segmentation, bias field correction and intensity standardization. Due to the automatic nature of this pre-processing, insufficient outcome can and does occur. For example, the bias field correction might fail, the skull-stripping can leave some skull tissue in the image or the intensity standardization can falsely skew the image’s histogram. A good classifier should be able to deal with such cases. For post-processing after voxel-wise classification, all connected binary objects with a size <1.5 ml are removed from the segmentation under the assumption that they constitute outliers, e.g. due to noise. The size corresponds to objects of a side length of less than 4 pixel at working resolution. The smallest lesion in the data set is 1.8 ml in volume. This procedure has previously been proven effective, especially to reduce the number of false-positives in the skull [8]. A schematic overview of the processing pipeline can be found in Fig 1.

### Classification methods

A total of 9 classification methods are evaluated and compared with each other in this study. The function and set-up of these classification methods is described in this section. If not noted otherwise, no effort has been undertaken to optimize their parameters for this segmentation problem. Instead, they were executed with their best-practice parameter values, i.e. the default parameters of the *scikit-learn* [13] toolkit.

#### Gaussian Naive Bayes

Naive Bayes classifiers approach the classification task with the “naive” assumption of independence between every pair of features. The Gaussian Naive Bayes classifier assumes the likelihood of the features to be Gaussian:

$$\begin{array}{c} P\left(x_{i}|y\right)=\frac{1}{\sqrt{2\pi \sigma_{y}^{2}}}\exp-\frac{{\left(x_{i}-\mu_{y}\right)}^{2})}{\sigma_{y}^{2}} \end{array}$$ (1),

where *x*_*i*_ is a dependent feature vector, *y* a class variable and the parameters *σ*_*y*_ and *μ*_*y*_ are estimated using maximum likelihood.

Even though GNB oversimplifies the reality, they have been found to perform surprising well in a number of real-world problems. Furthermore, GNB classifiers require only a small amount of training data, are parameter-free and train very fast. They are well researched, both from a theoretical [14] and empirical [15] point of view.

#### k-Nearest-Neighbors

The supervised k-Nearest-Neighbors [16] approach classifies testing samples by transferring the majority label of the *k* nearest training neighbors to the corresponding test case. Although multiple distance definitions have been proposed in the past, the Euclidean distance is used most commonly and also employed in this study.

kNN classifiers do not generalize from the training set, but simply store the training data. Comparable to GNB classifiers, k-Nearest-Neighbors models have been found to perform well for many real-world classification problems.

Besides the definition of the distance metric, the choice of *k* is crucial. Higher values for *k* reduce the influence of noise, whereas lower values lead to more distinct class boundaries. As an additional parameter, the training samples votes can be weighted by their distance. However, this feature was not used in this study to keep the method as simple as possible.

#### Generalized Linear Models

In a Generalized Linear Model, tissue infarction probability can, for example, be represented by the logistic function as typically used for biological applications:

$$\begin{array}{c} F\left(t\right)=\frac{e^{t}}{e^{t}+1} \end{array}$$ (2)

with *t* being a linear function of the input parameters *x*,

$$\begin{array}{c} t=\beta_{0}+\beta_{1}x_{1}+\ldots +\beta_{n}x_{n} \end{array}$$ (3)

The main advantages of the algorithm are the simplicity, comparably high speed for the training as well as for the testing phase, and possibility to investigate the different effects of the multiple input parameters on the outcome probability in terms of the *β* parameters. However, logistic regression models are also known to be unsuitable for inherently nonlinear problems.

#### Gradient Boosting classifier

Gradient Boosting (GB) classifiers describe a generalized boosting method to arbitrary differentiable loss functions. In case of the GB classifier implementation used in this study, this method is similar to decision forests in the sense that a large number of decision trees is trained. These weak classifiers are optimized at each stage to fit the negative gradient of the deviance (twice binomial negative log-likelihood) loss function, i.e. the steepest gradient descent. The learning rate regularization strategy proposed by Friedman et al. [17] is employed in this work, but not the bootstrapping strategy described in Friedman et al. [18], which would result in stochastic GBs.

GB classifiers are known to achieve a high predictive power and to be robust against outliers in output space. A severe drawback is their sequential nature, which leads to long training times. They can be considered a predecessor to decision forests.

GB classifiers required the definition of a number of hyper-parameters. In general, there is a trade-off between the learning rate and the number of estimators while the maximum tree depth should be kept small to allow faster training times. For this application, we chose to train 100 trees with a maximum depth of 20.

#### AdaBoost

AdaBoost (AB) [19] represents another well-known boosting method, where a sequence of weak learners is fitted to repeatedly modified versions of the training data. A weighted majority vote at application time is used to achieve the final class prediction. In contrast to decision trees, which utilize bootstrapping for this purpose, AdaBoost assigns individual weights to the training samples: The first weak classifier is trained on the uniformly weighted samples, then the weights are iteratively increased for training samples wrongly predicted in previous steps. Hence, difficult and complex training samples obtain a greater weight for later weak classifiers.

AdaBoost is often considered as one of the best out-of-the-box classifiers. Nonetheless, it is also known to be sensitive to noise and outliers, as it explicitly increases their influence.

The implementation used in this study employs decision tree stumps as weak classifiers. Important additional parameters are the number of estimators and the learning rate, which penalizes later classifiers. The first value was set to 100, the latter kept at its default value 1.0.

#### Random Decision Forests

Random Decision Forest classifiers [20] rate among the most recent and popular boosting methods and have proven their classification performance for difficult problems in many applications [6, 21]. Based on decision trees [22] as weak classifiers, they employ training set bagging [23] and random subspaces [24] to introduce a measure of randomness into the training.

RDF classifiers are inherently parallel and, hence, train very fast. The randomness avoids the training to get stuck at a local minimum, which improves the predictive accuracy and controls over-fitting.

While RDF classifiers depend on a number of parameters, such as the number of trees, the features considered at each split, and the maximum tree depth, they have been found to be easy to optimize [8, 21]. For this application, we chose to train 100 trees with a maximum depth of 20.

#### Extra Tree forests

Extra Tree (ET) forest classifiers are a variant of RDF introduced by Geurts et al. [25], which add an additional layer of randomness. Instead of searching for the optimal split, a random split threshold is used during the training of the decision trees. The implementation used in this work did not employ bootstrapping of the training data.

ETs have been found to decrease the variance at the cost of a bias even greater than it is the case for RDFs. Furthermore, they might show improved prediction for difficult classification problems with many inter-dependent features.

ET methods require the same parameters as RDF classifiers.

#### Convolutional Neural Networks

In recent benchmarks, neural networks present the winning solutions for various computer vision tasks like object detection, street number recognition and mitosis detection [26–28]. Convolutional Neural Networks [29] are a special form of neural networks that transform the input by repeated steps of convolution followed by pooling. The output of this feature extraction step forms the input to a classical fully connected neural network. The whole network including the kernels of the convolution is trained using back propagation.

By training their own feature extractors, CNNs can be easily applied to new problems. Their classification speed is comparable to other methods. However, their training time is considerably longer. Also the network’s architecture and multiple hyper parameters need to be chosen carefully for good results. In order to achieve a good generalization, a high training sample count, the convolutional architecture [30] and dropout layers [31] are recommended.

Contrary to the other methods presented in this paper, the CNN uses the raw image input instead of the manually designed features. Therefore, 10^7^ overlapping patches of 37*x*37*x*3 voxels are sampled from the training data in a uniform random manner and labeled according to the center voxel’s classification in the ground truth. For our experiments, the Caffe [32] framework is used. The network is built from three steps of convolution with rectified linear activation (RELU) and pooling, followed by one fully connected layers with RELU and one with softmax activation. The precise network architecture is described in Table 1. Learning was performed in a fully supervised manner using a batch size of 500, a learning rate of 0.0001, a weight decay of 0.004, and a momentum of 0.9.

**Table 1:** Convolutional neural network architecture.

Layer	Type	Maps and neurones	Kernel size
0	input	3 maps of 37 × 37 neurons	
1	convolution	100 maps of 35 × 35 neurons	3 × 3
2	pooling	100 maps of 18 × 18 neurons	2 × 2
3	convolution	150 maps of 16 × 16 neurons	3 × 3
4	pooling	150 maps of 8 × 8 neurons	2 × 2
5	convolution	150 maps of 6 × 6 neurons	3 × 3
6	pooling	150 maps of 3 × 3 neurons	2 × 2
7	fully connected	300 neurons	1 × 1
8	fully connected	2 neurons	1 × 1

The input is processed from the top to the bottom, where the two output neurons each represent one class. Rectified linear activation is used after each convolution and the first fully connected layer. The two final neurons are activated by a softmax function and can be interpreted as the probability of a particular input to belong to the respective class.

#### Tuned Extra Tree forests

To assess the upward potential of forest-based methods, we also included tuned Extra Trees forests (tunedET) in our set of classifiers. They are ET classifiers with tuned parameters for improved classification results as described in Maier et al. [8].

### Evaluation metrics

The evaluation of the nine classification techniques described above was conducted using three different metrics: (1) the dice metric (DM), which describes the volume overlap between two segmentations and is sensitive to the lesion size, (2) the average symmetric surface distance (ASSD), which denotes the average surface distance between two segmentations, and (3) the Hausdorff distance (HD), which is a measure of the maximum surface distance and is, hence, especially sensitive to outliers. Additionally, precision and recall values are reported for each classifier to assess over- and under-segmentation, respectively.

The DM is defined as

$$\begin{array}{c} DM=\frac{2|A\cap B|}{\left|A|+|B\right|} \end{array}$$ (4)

with *A* and *B* denoting the set of all voxels of ground truth and segmentation respectively. To compute the ASSD, we first define the average surface distance (ASD), a directed metric, as

$$\begin{array}{c} ASD\left(A_{S},B_{S}\right)=\frac{\sum_{a\in A_{S}}min_{b\in B_{S}}d\left(a,b\right)}{|A_{S}|} \end{array}$$ (5)

and then average over both directions to obtain the ASSD

$$\begin{array}{c} ASSD\left(A_{S},B_{S}\right)=\frac{ASD\left(A_{S},B_{S}\right)+ASD\left(B_{S},A_{S}\right)}{2} \end{array}$$ (6)

Here *A*_*S*_ and *B*_*S*_ denote the surface voxels of ground truth and segmentation respectively. Simmilar, the HD is defined as the maximum of all surface distances with

$$\begin{array}{c} HD\left(A_{S},B_{S}\right)=\text{max}\left\{\max_{a\in A_{S}}\min_{b\in B_{S}}d\left(a,b\right),\max_{b\in B_{S}}\min_{a\in A_{S}}d\left(b,a\right)\right\} \end{array}$$ (7)

The distance measure *d*(⋅) employed in both cases is the Euclidean distance, computed taking the voxel size into account. Finally, precision and recall are computed from true positive (*TP*), false positive (*FP*) and false negative (*FN*) voxels as

$$\begin{array}{c} precision=\frac{TP}{TP+FP} \end{array}$$ (8)

and

$$\begin{array}{c} recall=\frac{TP}{TP+FN} \end{array}$$ (9)

## Results

For the experiments, all methods were trained and evaluated with the leave-one-out evaluation schema, i.e. 36 cases were used for training and the remaining for testing in all possible combinations. At working resolution, the number of available voxels for training surpassed the ten million. To speed up training, only a sub-set of *n* = 500,000 of these were selected. For this purpose, we randomly sampled 500,000/36 ≈ 14,000 training voxels from each training case using stratified random sampling, i.e. keeping each cases lesion to background ratio intact. In a previous study [8] we have shown that using more than 100,000 samples did not significantly improve the results, and hence we chose here a larger value for *n* for an ample security margin. The exact positions of the randomly selected training voxels of each case that were used to generate the results presented in this article are available from the corresponding author on request. The CNN required another approach since it trains on the actual images and learns its own features.

For the experiments, we distinguish between two scenarios: (I) Under the assumption that a FLAIR image is almost always acquired for ischemic stroke assessment with MRI, the *flair* set of experiments is mono-spectral using only the FLAIR sequence. The results obtained for all classifiers are displayed in Table 2.

**Table 2:** *Flair* scenario.

Classifier	DM [0, 1]	HD (mm)	ASSD (mm)	Prec. [0, 1]	Rec. [0, 1]	Cases	Traintime
100 Nearest Neighbors	0.54**± 0.20	36.52± 22.4	7.07**± 4.25	0.82	0.45	34/37	5s
10 Nearest Neighbors	0.56**± 0.20	36.47± 25.1	6.58*± 4.01	0.82	0.46	35/37	5s
5 Nearest Neighbors	0.58**± 0.18	39.72*± 27.4	6.80*± 4.35	0.79	0.51	36/37	5s
AdaBoost	0.60*± 0.19	39.28*± 27.3	7.42*± 6.77	0.70	0.61	35/37	7m
Extra Trees	0.64**± 0.19	29.49± 18.5	5.29± 3.94	0.84	0.57	35/37	3m
Gaussian Naive Bayes	0.48**± 0.22	69.86**± 26.7	14.82**± 8.16	0.44	0.78	36/37	1s
Generalized Linear Model	0.44**± 0.25	38.77*± 21.3	8.54**± 5.76	0.87	0.34	32/37	2m
Gradient Boosting	0.63**± 0.18	32.72± 23.2	5.93± 5.28	0.72	0.62	35/37	12h
Random Decision Forest	**0.67**± 0.18	**28.16**± 20.7	**4.89**± 3.63	0.82	0.62	35/37	6m
Convolutional Neural Network	0.67± 0.18	29.64± 24.6	5.04± 5.28	0.77	0.64	35/37	2h

(II) In the clinical routine, the acquisition of some MRI sequences can be skipped due to various reasons. Our second setting constitutes a *besteffort* approach to handle the sparsity in the available sequences for each case. If available, the T1w and/or DWI sequences are used in addition to the FLAIR imaging information, which led to the requirement of training multiple dependent classifiers. I.e. a specialized classifier is trained on all cases with FLAIR sequences (*n* = 37) and employed to segmented FLAIR-only test cases (*n* = 16); a FLAIR+T1w classifier is trained on all corresponding cases (*n* = 21) and employed to segment cases with a FLAIR and a T1w sequence available (*n* = 7); the same applies to FLAIR+T1w+DWI (*n* = 14 for both, training and testing). The results obtained with this *besteffort* configuration are given in Table 3.

**Table 3:** *Besteffort* scenario.

Classifier	DM [0, 1]	HD (mm)	ASSD (mm)	Prec. [0, 1]	Rec. [0, 1]	Cases
100 Nearest Neighbor	0.61**± 0.21	38.10**± 26.5	6.10**± 4.03	0.82	0.55	34/37
10 Nearest Neighbor	0.63**± 0.21	35.85**± 26.1	5.62**± 3.96	0.82	0.56	36/37
5 Nearest Neighbor	0.63**± 0.19	38.68**± 28.6	6.00**± 4.40	0.78	0.59	36/37
AdaBoost	0.69± 0.16	32.65*± 25.5	5.60± 5.84	0.73	0.68	34/37
Extra Trees	0.70**± 0.19	23.18± 15.4	3.98**± 3.56	0.85	0.64	35/37
Gaussian Naive Bayes	0.54**± 0.20	71.48**± 22.9	12.01**± 5.36	0.47	0.82	36/37
Generalized Linear Model	0.55**± 0.27	32.44**± 23.8	6.38**± 5.77	0.90	0.47	34/37
Gradient Boosting	0.68**± 0.17	25.83± 19.0	3.95± 2.89	0.79	0.65	35/37
Random Decision Forest	**0.72**± 0.17	**22.35**± 15.8	**3.67**± 3.35	0.84	0.68	35/37
tuned Extra Trees	0.73*± 0.18	21.48± 12.0	3.49± 2.76	0.84	0.69	35/37

In both tables, the best-performing method for each evaluation measure is marked in bold. Significant differences to this best-performing method computed with student’s paired t-test are marked with a star (*) for a confidence interval of 95% (*p* < 0.05) and two stars (**) for a confidence interval of 99% (*p* < 0.01). Nominal p-values are reported without correction for multiplicity. Note that the tunedET were exempt from the selection of the best-performing method, as they were tuned for performance. The full results for each classifier and case can be found in S1 File. It should be noted that some methods failed completely for certain cases (i.e. achieved a DM of 0). The corresponding datasets were excluded from the calculation of the average values for all methods to enable a direct and fair comparison.

The inter-observer differences between the two expert segmentations are given in Table 4.

**Table 4:** Inter-observer score.

DM [0, 1]	HD (mm)	ASSD (mm)	Prec. [0, 1]	Rec. [0, 1]
0.80	15.79	2.03	0.73	0.911

GTG vs. GTL

To assess each methods dependency on the ground truth, Table 5 shows respective cross validations for selected evaluation measures.

**Table 5:** Dependency on training ground-truth.

	GTG↦GTG		GTG↦GTL		GTL↦GTL		GTL↦GTG	
Classifier	DM [0, 1]	ASSD (mm)	DM [0, 1]	ASSD (mm)	DM [0, 1]	ASSD (mm)	DM [0, 1]	ASSD (mm)
Generalized Linear Model	0.55	6.38	0.58	5.77	0.57	5.84	0.52	6.66
Random Decision Forest	0.72	3.67	0.72	3.46	0.72	3.31	0.69	3.92
tuned Extra Trees	0.73	3.49	0.72	3.28	0.73	3.21	0.70	3.81

Results for selected methods on different combination of training and testing ground truth sets in *besteffort* scenario.

**Fig 2:**
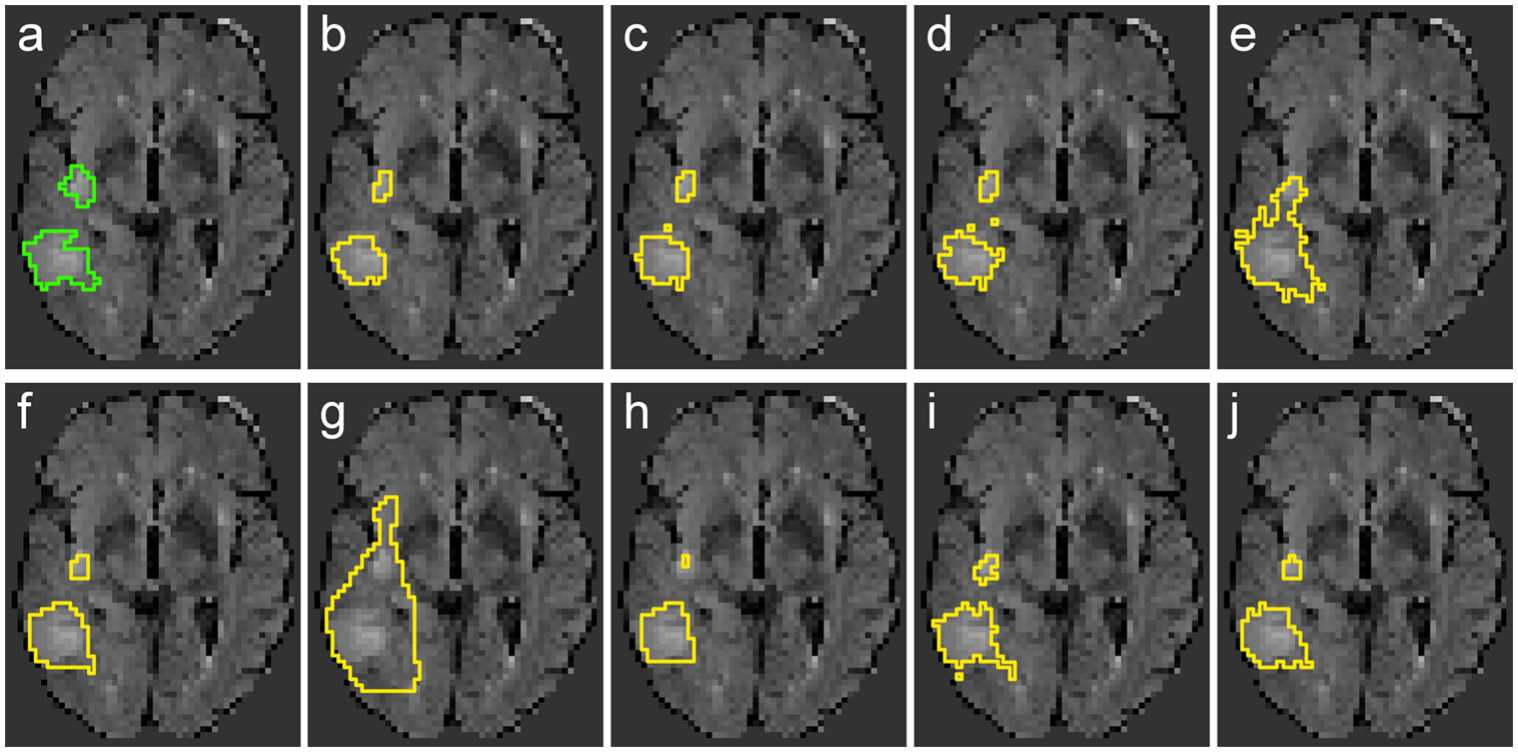
Results for case 21. Slice 21 with *besteffort* scenario trained on GTG. (a) ground-truth (GTG). (b) 100NN. (c) 10NN. (d) 5NN. (e) AdaBoost. (f) ET. (g) GNB. (h) GLM. (i) GB. (j) RDF.

**Fig 3:**
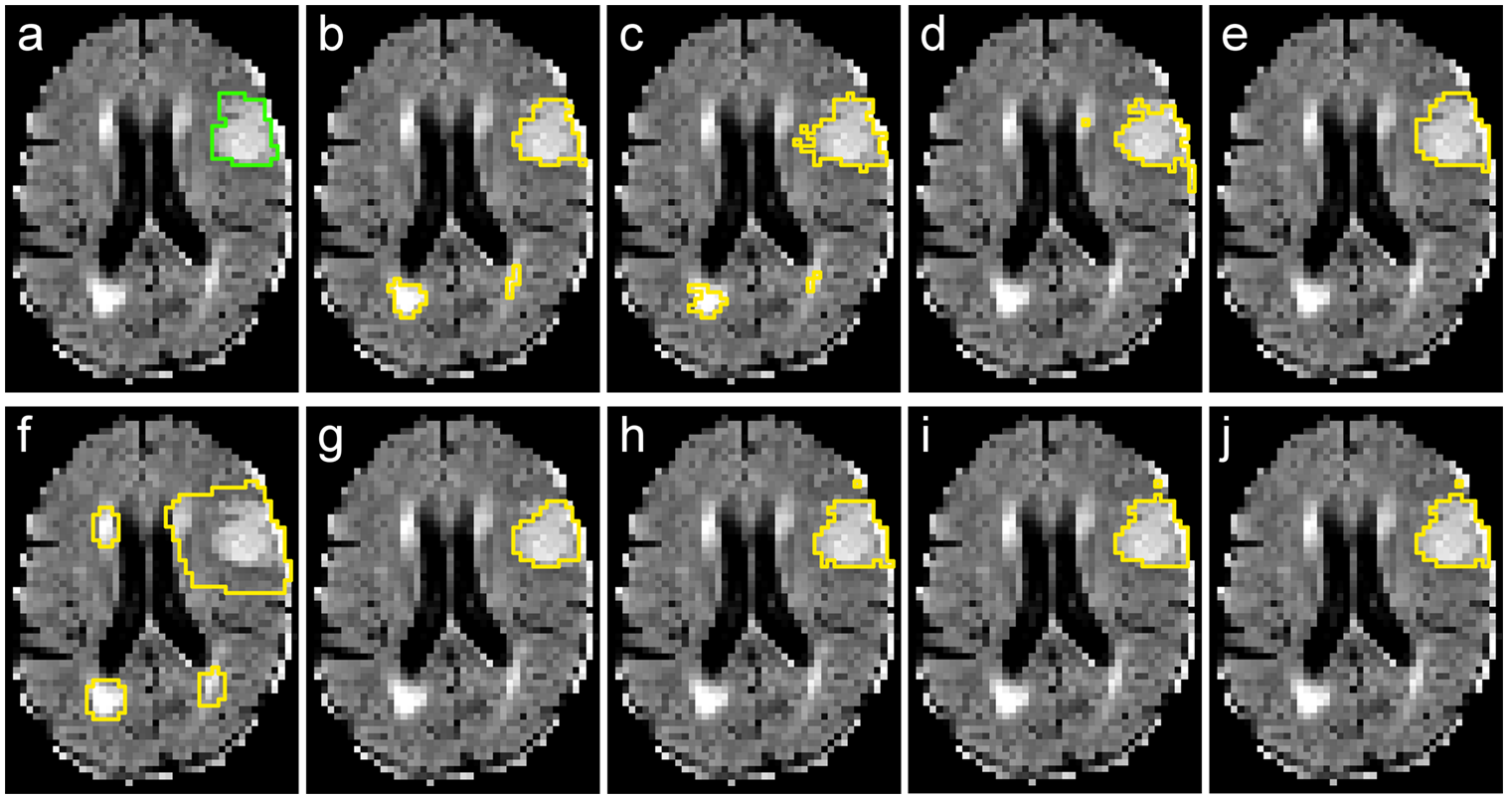
Results for case 04. Slice 30 with *besteffort* scenario trained on GTG. Note the presence of other white matter hyperintensities. (a) ground-truth (GTG). (b) 100NN. (c) 5NN. (d) AdaBoost. (e) ET. (f) GNB. (g) GLM. (h) GB. (i) RDF. (j) tunedET.

Visual results for a rather simple case are presented in Fig 2, and for a more complicated dataset with other white matter hyperintensities present in Fig 3

Case-wise results for all methods can be found in S1 File on the Evaluation Dataset and be used to reconstruct the means and statistical significancies.

**Fig 4:**
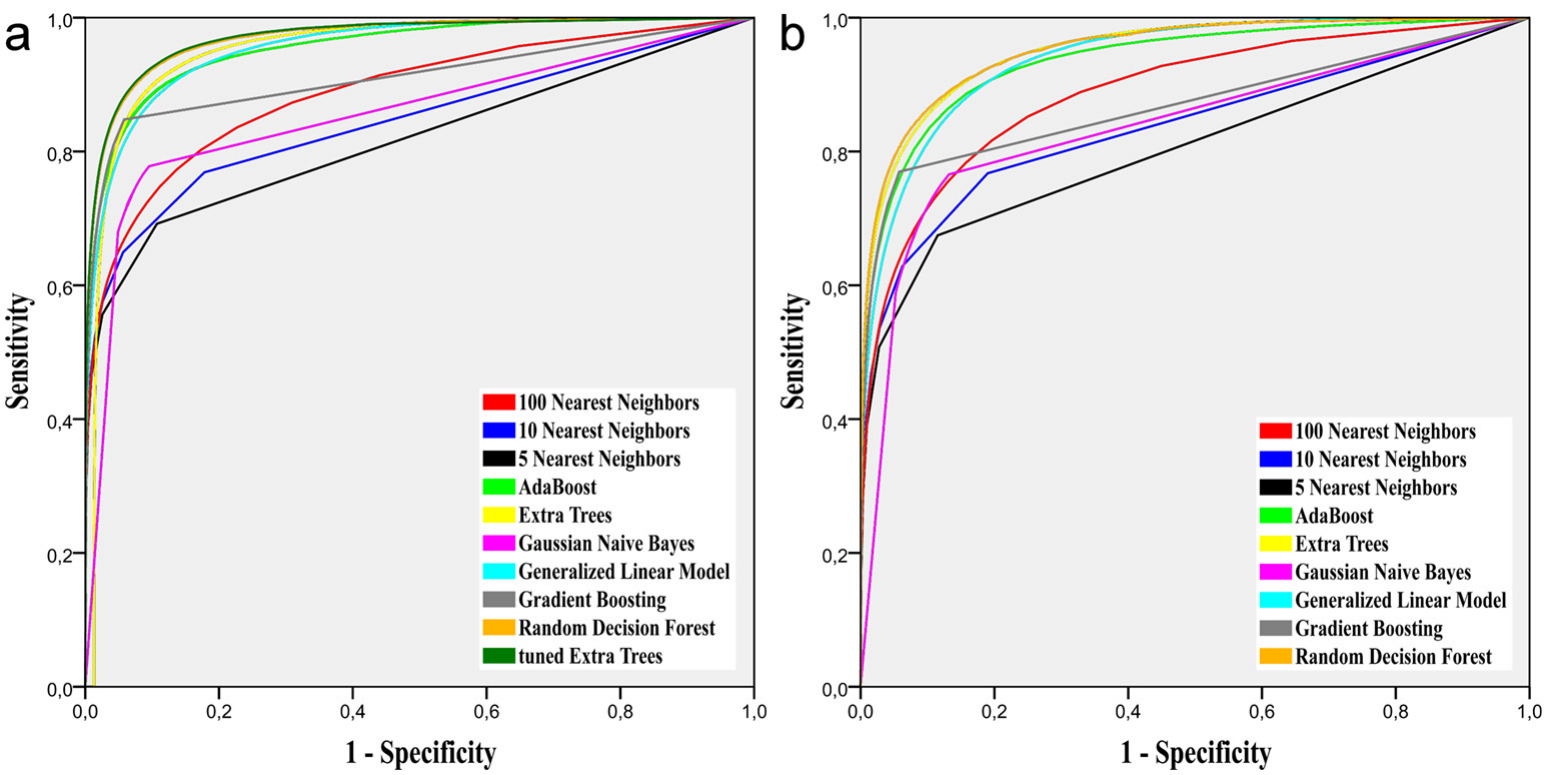
ROC curves for both evaluation scenarios computed over the GTG ground truth. (a) *besteffort* scenario. (b) *flair* scenario.

To evaluate the different algorithms theoretical optimal performance optained by thresholing the a-posteriori class probability maps, Fig 4 shows the Receiver Operating Characteristic (ROC) curves. These have been obtained for both evaluation scnearios on the GTG ground truth set. Some associated Area Under Curve (AUC) values for the *besteffort*-scenario are: tunedET = .97, RDF = .97, GLM = .96, ET = .95, AdaBoost = .95, GB = .91, 100NN = .89

## Discussion

### Method-specific interpretation

Overall, the results revealed that the RDF classifier consistently and significantly outperformed all other non-tuned classifiers for all ground truth sets and scenarios. Adding their relatively fast training times, RDF classifiers can, hence, be considered the best candidate for further method development as well as the baseline other classification-based lesion segmentations should be compared with.

The ET classifier performed similar well and stable, but also increases the bias considerably, as indicated by the high precision values. The forest related GB classifier led to the overall third-best results. However, the excessively long training times of this classification method render it unsuitable for rapid development and testing. The results of AdaBoost, the last in the group of ensemble methods, showed a clear upward step from mono- to multi-spectral input data. This might be attributed to the better outlier avoidance in the *besteffort* pre-processing.

The simple kNN classifiers led to the best results among the non-ensemble classifiers evaluated in this study. While fast to train, they showed an overly high precision at the cost of recall, hinting towards complicated decision borders for the classification problem. Next are the GLM results, that appear to fail finding a linear decision border in the *flair* scenario, which is a known drawback of this classifier. However, the results of the GLM classifier showed an impressive gain when employing multi-spectral data, as a higher dimensional feature space enables more flexibility regarding the border placement. GNB, the simple and parameter-free classifier, scores last and clearly leads to an over-segmentation of the lesions. All of these findings are supported by the visual evaluation (see Figs 2 and 3).

The CNNs perform nearly head to head with the RDFs, but care must be taken interpreting the results, as they were not obtained using the same feature set. Rather, the comparison must be conducted in terms of potential. The results for the tuned ETs give an idea of the expectable gain for the ensemble methods, which is significant (at *p* < 0.05), but clearly limited. The CNN method, on the other hand, is highly configurable, which, taken together with the intrinsic feature detection, may bare high potential for even better multi-spectral segmentation results. Drawbacks are the black-box character, the difficult parameter tuning, the high system requirements and of course the large training times.

The ROC curves (Fig 4) of the tested algorithms and their associated AUC values provide a measure for each method’s performance for the ideal cut-point of the a-posteriori class probability maps. The results supports above observations that the ensemble methods perform generally better. An exception is the GLM, whose curve is simmilar to the AdaBoost approach. For an ideal cut-point of the a-posteriori class probability maps, the GLM would rate directly after the RDF and tunedET, on the same level as AdaBoost and the ET algorithms. For the *flair*-scenario, they fall behind the ET.

### Failed cases

**Fig 5:**
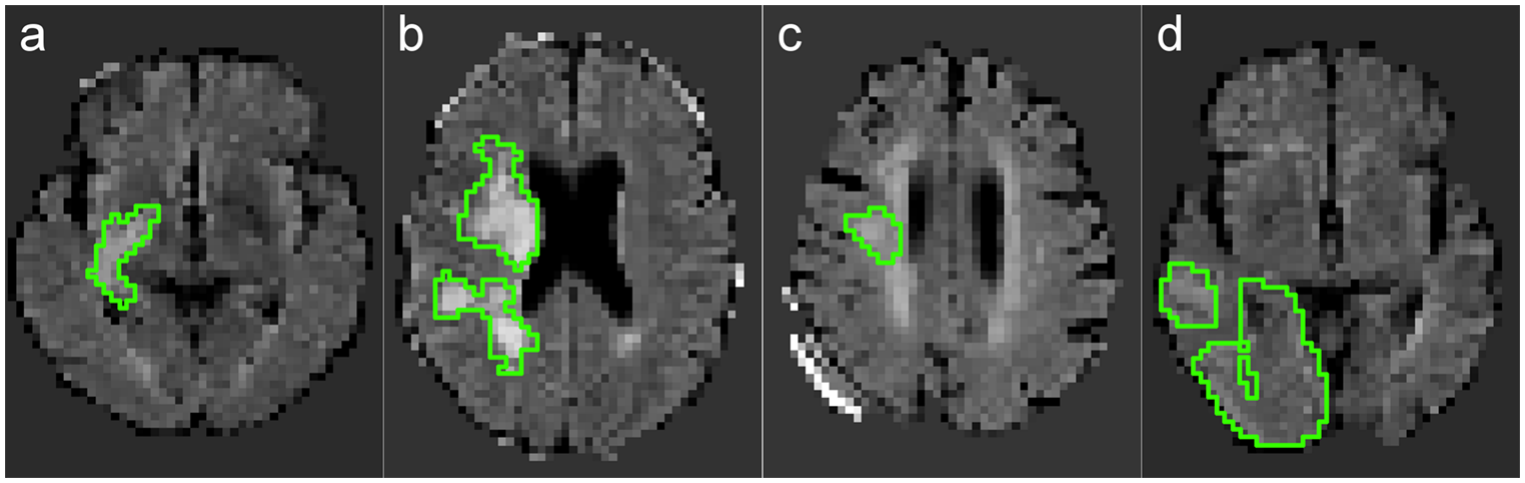
Cases failed by at least one classifier. (a) 09/24 ground-truth (GTG). (b) 11/29 ground-truth (GTG). (c) 39/36 ground-truth (GTG). (d) 41/24 ground-truth (GTG).

**Fig 6:**
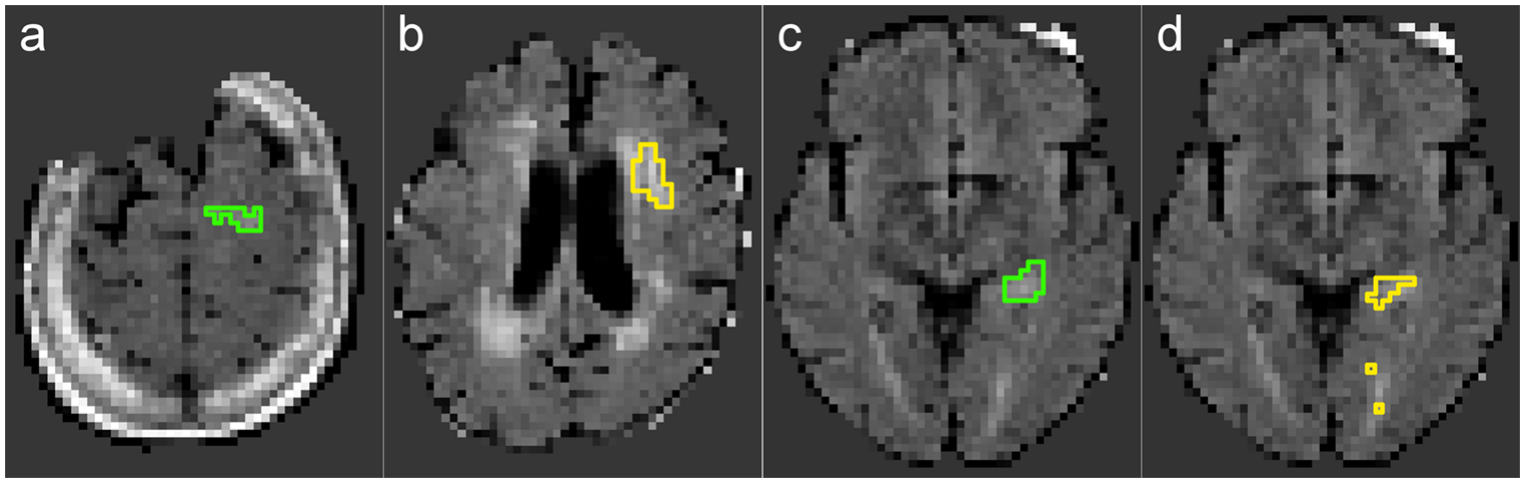
Worst two cases. See text for description. (a) 37/44 ground-truth (GTG). (b) 37/32 tunedET. (c) 44/24 ground-truth (GTG). (d) 44/24 CNN.

For some of the cases, at least one classifier failed to produce valid results (i.e. a DM>0). These were excluded from the computation of the evaluation measure means in Tables 2 and 3, and are shown in Figs 5 and 6.

The cases 09, 11 and 41 posed problems to the GLM, which might be attributed to their unusual high (case 11) respectively low (cases 09 and 41) hyperintensities inside the lesion area. In general, the linear model of the GLM did not adapt well to the complexity of the task and produced with *n* = 5 the largest number of failed cases, followed by the 100NN approach (*n* = 3), while all others did not fail in more than *n* = 2 cases.

Most notably among the failed cases are 37 and 44. For 37, all methods failed to produce a valid segmentation. Taking a look at the ground truth (Fig 6(a)), we observe a small lesion in the superior regions of only minor hyperintensity. A typical failed segmentation, as displayed in Fig 6(b), assumed the lesion to be among the numerous periventricular white matter hyperintensities. For case 44, some methods managed to segment part of the lesion (Fig 6(d)), but the maximum DM value reached has been 0.21. This lesion is very small, periventricular and of low hyperintensity.

For both cases, only the FLAIR sequence has been available, missing potentially relevant information from the other MRI sequences which might have facilitated the segmentation task.

### Visual interpretation

**Fig 7:**
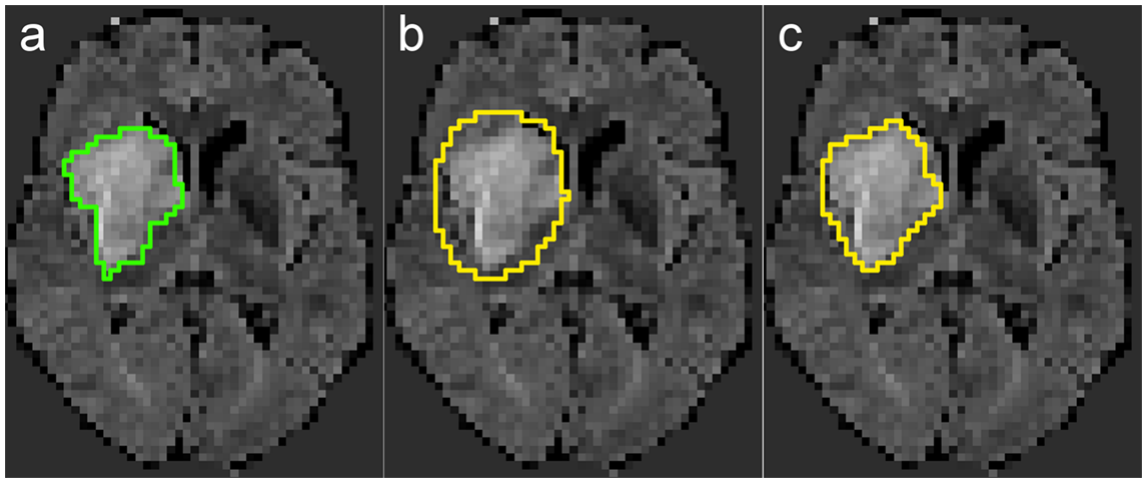
Best overall case 36 and the worst (GNB, DM = 0.61) as well as best (ET, DM = 0.86) result obtained over all methods. (a) 36/27 ground-truth (GTG). (b) 36/27 GNB. (c) 36/27 ET.

With an average DM of 0.80 over all the different methods tested, case 36 can be considered an easy case with a standard deviation as low as 0.07. Fig 7 depicts its ground truth as well as the best and worst result obtained.

The image displays a single, large and homogeneously hyperintense lesion. Differences between the methods stem mainly from classifier specific tendencies, such as the over-segmentation of the GNB.

**Fig 8:**
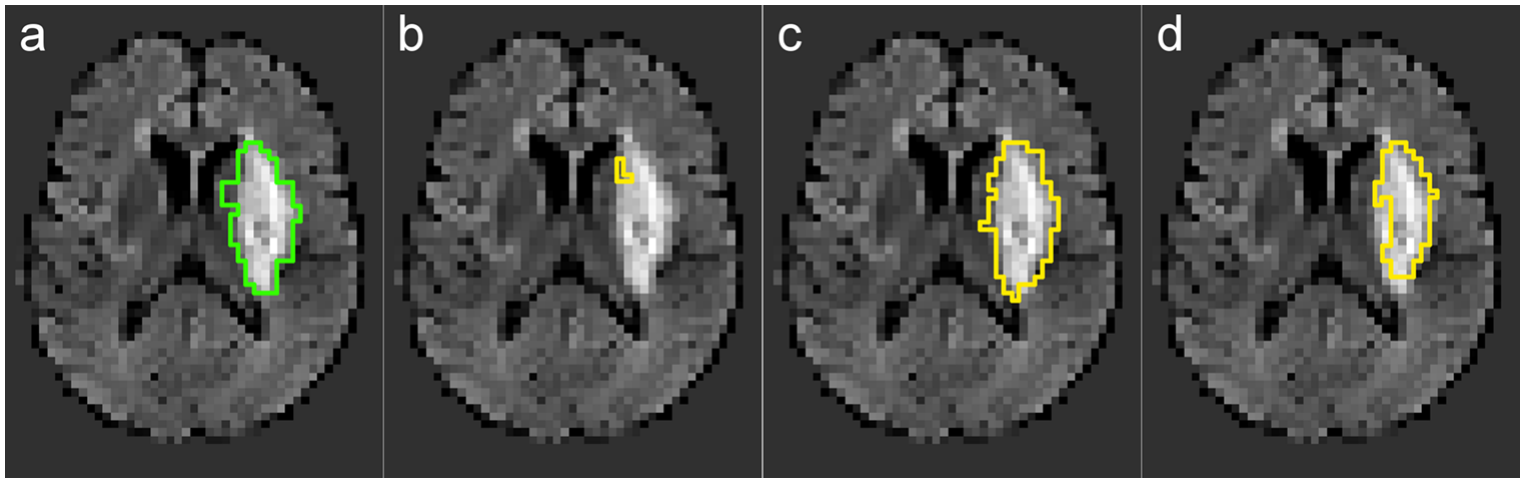
Case with low agreement between methods in *flair* scenario. (a) 18/28 ground-truth (GTG). (b) 18/28 AdaBoost. (c) 18/28 CNN. (d) 18/28 ET.

Another case to take a close look at is 18, for which the largest standard deviation over all methods has been obtained (DM = 0.55±0.31). As can be seen in Fig 8, the lesion is clearly outlined and strongly hyperintense, hence the task should be an easy one.

And such it seems when looking at the DM results of the CNN (0.85) and the 5NN respectively 10NN classifiers (both 0.81). Most other methods performed acceptably with values around 0.60. But on the lower end, we have the GNB (0.16), AdaBoost (0.08) and the GLM (0.00). These failures might be attributed to the unusual high intensity values inside the lesion paired with the low extrapolation and generalization abilities of the latter methods.

Under the *besteffort* approach, when the T1 and DWI sequences are equally considered, the inter-method standard deviation for case 18 drops to 0.09, signaling greater agreement. In general, when comparing the *besteffort* to the *flair* scenario, we reach the conclusion that it is better to use all available information than only the least common denominator.

### Inter-observer variability

The inter-observer differences (Table 4) are relatively high, which underlines the difficulties associated with this segmentation task and emphasizes the need for an automatic and, above all, reproducible segmentation method. Precision and recall reveal the GTL ground truth set to contain consistently smaller lesion masks. However, the manual segmentation is still superior compared to the automatic segmentation as there was no complete disagreement between the raters (i.e. a DM value of 0) for any of the datasets used in this study.

The methods ranking order is stable for all ground-truth sets and scenarios, i.e. all of them adapt well to the underlying model. Using one ground truth set as training and the other as testing did not lead to considerable performance differences. Therefore, it may be argued that all direct comparisons of the methods used in this study are sound, independently of the ground truth set employed.

### Comparison with results from literature

The overall best segmentation results were achieved by the tuned Extra Tree forests. The quantitative results of this method as well as the un-tuned ET, RDF and CNN accuracies, are superior compared to all previously reported results in literature. Wilke et al. [5] reported a DM of 0.60 for their semi-automatic and 0.49 for their automatic approach. Hevia-Montiel et al. [33] reported 0.54±0.18 and Seghier et al. [3] even 0.64±0.10, with only eight real cases used for evaluation. Mitra et al. [6] achieved an average DM of 0.60±0.13 and ASSD of 3.06±3.17 mm with RDFs. However, it should be noted that these comparisons are not truly valid, as different datasets and different ground truth segmentations were used for evaluation. Regrettably, no publicly available dataset existed to compare follow-up ischemic stroke lesion segmentation methods before 2015.

### Characteristics of ischemic stroke lesion segmentation

The results of this study enable us to make some assumptions about the nature of the ischemic stroke lesion segmentation problem. First, the rather low inter-observer agreement demonstrates the difficulty of the segmentation problem. Considering the subsequent uncertainty in the ground truth lesion masks, the training set can be expected to be noisy and outlier-ridden, an observation which is supported by the low performance of the noise sensitive AdaBoost classifier.

The results of the GLM classifier dispute the existence of a linear separation border between lesion and other tissue, even in the multi-spectral case. Hence, the classification problem can be considered non-linear. The employed features seem to be neither completely dependent nor completely independent, in which cases one would have expected better GNB results [15]. Furthermore, the comparably poor results obtained for the kNNs show that the different features are not equally important, one of the main kNN assumptions.

Finally, the good performance of the RDF classifier hint towards a high variance and low bias of the problem, although not unbalanced enough to justify the use of the ET classifier.

To sum up, the ischemic stroke lesion classification problem is clearly a difficult one with many challenging characteristics.

## Supporting Information

S1 File Detailed evaluation results. Case by case results of the leave-one-out cross-validation on all 37 cases with varying MRI sequences over all classifiers.
(ZIP)
