# Correction: Classifiers for Ischemic Stroke Lesion Segmentation: A Comparison Study

**DOI:** 10.1371/journal.pone.0149828

**Published:** 2016-02-12

**Authors:** Oskar Maier, Christoph Schröder, Nils Daniel Forkert, Thomas Martinetz, Heinz Handels

There is an error in the Conclusions section of the manuscript. The entire Conclusions section was not included. The Conclusions section should read:

In this work, nine different classifiers were used for ischemic stroke lesion segmentation from brain MRI images and evaluated using different ground truth sets and scenarios. Based on the results of this study, it seems justified to recommend RDF classifiers as the basis for method development, as they are fast, stable, and robust. Within this context, alternative features, as well as better pre- and post-processing methods should be investigated. Ischemic stroke lesion segmentation is a difficult problem with uncertain ground truth and a strong dependency on the pre-processing methods. Hence, improvements in this area are as important as developing better classifiers and features.

While the obtained RDF classification results outperform all previously published methods, human observer accuracy is not yet reached and ischemic stroke lesion segmentation remains a complicated problem. Apart from RDF classifiers, convolutional neural networks appear to hold more potential for improvement and should be employed in use-cases where accuracy is considered more important than speed, usability, and ease-of-configuration.

While this study compared different classifier solutions for ischemic stroke lesion segmentation in detail, the results have been obtained under the premise of a single type of pre-processing and a fixed set of features (except in the case of the CNN). It would be desirable to investigate the influence of different features and pre-processing decisions on the segmentation results.

Furthermore, the presented methods have been devised and implemented by a single team of researchers. An open-for-all comparison, as e.g. the scheduled ISLES Challenge at the forthcoming MICCAI 2015 conference, will provide a greater insight in the difficult problem of stroke lesion segmentation.

There is an error in Table 2 as well as Table 3. The caption for each table is missing. The authors have provided the corrected version here.

**Table 2:** Flair scenario

Classifier	DM [0, 1]	HD (mm)	ASSD (mm)	Prec. [0,1]	Rec. [0,1]	Cases	Traintime
100 Nearest Neighbors	0.54**± 0.20	36.52± 22.4	7.07**± 4.25	0.82	0.45	34/37	5s
10 Nearest Neighbors	0.56**± 0.20	36.47± 25.1	6.58*± 4.01	0.82	0.46	35/37	5s
5 Nearest Neighbors	0.58**± 0.18	39.72*± 27.4	6.80*± 4.35	0.79	0.51	36/37	5s
AdaBoost	0.60*± 0.19	39.28*± 27.3	7.42*± 6.77	0.70	0.61	35/37	7m
Extra Trees	0.64**± 0.19	29.49± 18.5	5.29± 3.94	0.84	0.57	35/37	3m
Gaussian Naive Bayes	0.48**± 0.22	69.86**± 26.7	14.82**± 8.16	0.44	0.78	36/37	1s
Generalized Linear Model	0.44**± 0.25	38.77*± 21.3	8.54**± 5.76	0.87	0.34	32/37	2m
Gradient Boosting	0.63**± 0.18	32.72± 23.2	5.93± 5.28	0.72	0.62	35/37	12h
Random Decision Forest	0.67± 0.18	28.16± 20.7	4.89± 3.63	0.82	0.62	35/37	6m
Convolutional Neural Network	0.67± 0.18	29.64± 24.6	5.04± 5.28	0.77	0.64	35/37	2h

Trained with GTG, evaluated on GTG, average computed over 31/37 cases, stars denote significant difference to best-performing method (in bold) with ** = p < 0.01 and * = p < 0.05, train-times given for a single training round, value after ± denotes the standard deviation.

**Table 3:** Besteffort scenario

Classifier	DM [0, 1]	HD (mm)	ASSD (mm)	Prec. [0,1]	Rec. [0,1]	Cases
100 Nearest Neighbor	0.61**± 0.21	38.10**± 26.5	6.10**± 4.03	0.82	0.55	34/37
10 Nearest Neighbor	0.63**± 0.21	35.85**± 26.1	5.62**± 3.96	0.82	0.56	36/37
5 Nearest Neighbor	0.63**± 0.19	38.68**± 28.6	6.00**± 4.40	0.78	0.59	36/37
AdaBoost	0.69± 0.16	32.65*± 25.5	5.60± 5.84	0.73	0.68	34/37
Extra Trees	0.70**± 0.19	23.18± 15.4	3.98**± 3.56	0.85	0.64	35/37
Gaussian Naive Bayes	0.54**± 0.20	71.48**± 22.9	12.01**± 5.36	0.47	0.82	36/37
Generalized Linear Model	0.55**± 0.27	32.44**± 23.8	6.38**± 5.77	0.90	0.47	34/37
Gradient Boosting	0.68**± 0.17	25.83± 19.0	3.95± 2.89	0.79	0.65	35/37
Random Decision Forest	0.72± 0.17	22.35± 15.8	3.67± 3.35	0.84	0.68	35/37
tuned Extra Trees	0.73*± 0.18	21.48± 12.0	3.49± 2.76	0.84	0.69	35/37

Trained with GTG, evaluated on GTG, average computed over 33/37 cases, stars denote significant difference to best-performing method (in bold) with ** = p < 0.01 and * = p < 0.05, value after ± denotes the standard deviation.
